# Establishment of Apomixis in Diploid F_2_ Hybrids and Inheritance of Apospory From F_1_ to F_2_ Hybrids of the *Ranunculus auricomus* Complex

**DOI:** 10.3389/fpls.2018.01111

**Published:** 2018-08-03

**Authors:** Birthe H. Barke, Mareike Daubert, Elvira Hörandl

**Affiliations:** Department of Systematics, Biodiversity and Evolution of Plants, Albrecht-von-Haller Institute for Plant Sciences, University of Göttingen, Göttingen, Germany

**Keywords:** apospory, developmental biology, endosperm balance, FCSS, gametophytic apomixis, hybrid, polyploidy, *Ranunculus*

## Abstract

Hybridization and polyploidization play important roles in plant evolution but it is still not fully clarified how these evolutionary forces contribute to the establishment of apomicts. Apomixis, the asexual reproduction via seed formation, comprises several essential alterations in development compared to the sexual pathway. Furthermore, most natural apomicts were found to be polyploids and/or hybrids. The *Ranunculus auricomus* complex comprises diploid sexual and polyploid apomictic species and represents an excellent model system to gain knowledge on origin and evolution of apomixis in natural plant populations. In this study, the second generation of synthetically produced homoploid (2*x*) and heteroploid (3*x*) hybrids derived from sexual *R. auricomus* species was analyzed for aposporous initial cell formation by DIC microscopy. Complete manifestation of apomixis was determined by measuring single mature seeds by flow cytometric seed screen. Microscopic analysis of the female gametophyte formation indicated spontaneous occurrence of aposporous initial cells and several developmental irregularities. The frequency of apospory was found to depend on dosage effects since a significant increase in apospory was observed, when both F_1_ parents, rather than just one, were aposporous. Other than in the F_1_ generation, diploid *Ranunculus* F_2_ hybrids formed B_III_ seeds and fully apomictic seeds. The results indicate that hybridization rather than polyploidization seems to be the functional activator of apomictic reproduction in the synthetic *Ranunculus* hybrids. In turn, at least two hybrid generations are required to establish apomictic seed formation.

## Introduction

Apomixis in Angiosperm plants is, by definition, seed formation via asexual reproduction, resulting in clonal, maternal offspring (Nogler, 1984a). Gametophytic apomixis, which is the focus of our study, combines two steps: (1) apomeiosis, i.e., the formation of an unreduced embryo sac, and (2) parthenogenesis, i.e., the development of an unfertilized egg cell into an embryo. Almost all apomictic plants are polyploids and/or hybrids but the role of these processes for establishment of apomixis is still not well-understood. There is evidence that the functional establishment of apomixis is not exclusively ploidy-dependent but an important factor in increasing and optimizing related gene expression (Quarin et al., 2001; Bicknell and Koltunow, 2004; Comai, 2005). A reason for the importance of polyploidy in apomictic plants can be conjectured by gene dosage effects, which state that haploid gametophytes abort due to recessive lethal effects of apomixis-controlling genetic factors (Nogler, 1982, 1984a,b). This assumption is supported by the rarity of diploid apomicts but a few exceptions are Scandinavian *Potentilla argentea* biotypes, diplosporous *Boechera* species (Müntzing, 1928; Böcher, 1951; Sharbel et al., 2009), *Paspalum* and *Ranunculus kuepferi* individuals (Ortiz et al., 2013; Schinkel et al., 2016, 2017). However, emergence of apomixis is not only achieved by ploidy but could be also an effect of hybridization (Asker and Jerling, 1992). Often hybridization of sexual plants leads to severe disturbances influencing genetic and epigenetic composition or meiotic cell division that can result in progeny with reduced fitness (Carman, 1997; Rieseberg et al., 1999; Comai, 2005). Disturbances are thought to be attenuated by the mentioned allopolyploidization, which in turn might lead to asynchronous gene expression due to stabilization and inheritance of genomic changes (Mogie, 1992; Carman, 1997). One possibility to get away from hybrid sterility is the switch to apomictic reproduction as hypothesized by Darlington (1939).

This switch is still not well-understood but many hypotheses have been developed, which involve several different molecular scenarios like genetic control mechanisms or epigenetic regulation. One popular hypothesis claims that heterochronic expression of sexual reproduction genes, which is caused by hybridization, is the trigger for apomictic seed formation (Carman, 1997; Sharbel et al., 2009, 2010). This idea is supported by recent findings of Hojsgaard et al. (2014), who discovered severe changes in the timing of megagametogenesis in synthetic *Ranunculus auricomus* F_1_ hybrids. In early studies, it was assumed that apomixis is inherited as single dominant trait and maybe as only one gene (e.g., Nogler, 1984a; Savidan, 1992). More recent studies have shown that important apomictic characteristics such as apomeiosis, parthenogenesis and fertilization-independent endosperm formation seem to be controlled by several independent loci (e.g., Schallau et al., 2010; Ogawa et al., 2013). The developmental pathways of *Hieracium* apomicts support these findings because mutant plants were able to return to sexuality, when lacking the apospory locus (Catanach et al., 2006; Koltunow et al., 2013). Although, gene expression studies were carried out, no connection between apomixis and certain gene clusters were identified, but it was determined that apomixis often co-segregates with a block of gene-poor heterochromatin (Huo et al., 2009; Ochogavía et al., 2011; Grimanelli, 2012). Apomictic reproduction in angiosperm plants is a heritable and facultative process probably regulated by differently expressed genes responsible for controlling sexual development or it might be the result of reversible, epigenetic silencing (Hand and Koltunow, 2014). Amongst others, Carman (1997) proposed that the switch to asexual seed formation is triggered by gene duplication subsequently followed by changes in epigenetic gene expression (e.g., Koltunow, 1993). Today, it is verified that hybridization and polyploidization can result in altered epigenetic regulations as well as genetic changes in plants (Comai, 2005). DNA modifications such as methylations or RNA interference are heritable and do not affect DNA sequences (Jaenisch and Bird, 2003) but such dosage effects might be the activator of apomictic development after hybridization or polyploidization events (Ozias-Akins and van Dijk, 2007). Thus, epigenetic regulation and reprogramming of plant development can be important factors for apomixis activation (Grimanelli, 2012). Identification of apomixis loci is difficult because recombination is often suppressed in these regions, which might be caused by allelic divergence (Hand and Koltunow, 2014).

The *R. auricomus* complex consists of mainly apomictic polyploid species but additionally a few di- and tetraploid obligate sexual species (*R. carpaticola, R. cassubicifolius*, and *R. notabilis*) are known (Hörandl and Gutermann, 1998; Paun et al., 2006; Hörandl et al., 2009; Hojsgaard et al., 2014). Sexually reproducing species were found to be self-incompatible, while the apomicts, like typical allopolyploids, were characterized as self-fertile (Hörandl, 2008). In *R. auricomus* plants gametophytic apomixis was described already by Nogler (1984a,b), starting with aposporous formation of an unreduced embryo sac from a somatic nucellar cell in short proximity to a meiotically developed megaspore tetrad or embryo sac that subsequently aborts. The embryo is formed parthenogenetically, whereas successful endosperm development usually requires fertilization of the polar nuclei (pseudogamy; Koltunow and Grossniklaus, 2003; Koltunow et al., 2011). Asexual *Ranunculus* taxa are not obligate apomicts because they still comprise, to some extent, the capacity to reproduce sexually (Nogler, 1984a,b; Hojsgaard et al., 2014; Klatt et al., 2016).

Although apomixis has been studied for more than 100 years now, it is still unclear, how the effective switch toward apomixis in natural plant populations is achieved. More specifically, the specific effects of hybridity vs. polyploidy on developmental pathways are unclear and difficult to entangle in natural allopolyploid apomicts. This study wants to shed light on the developmental events right upon hybridization vs. polyploidy in synthetic F_2_ plants of the *R. auricomus* complex as a potential cause of apomixis. Hojsgaard et al. (2014) analyzed the corresponding parental F_1_ hybrid generation to the plants used in this study and described first evidence of spontaneous apospory and developmental asynchrony in diploid and triploid hybrid *Ranunculus* gametophytes. However, functional apomictic seeds were only produced in polyploids, at very low frequencies. Here, we investigate F_2_ hybrid plants generated by manual crossing, where either both parents or one parent had apospory before (Hojsgaard et al., 2014). Since hybridization often is connected to allopolyploidization, which was also shown for natural hybrids of the *R. auricomus* complex (Paun et al., 2006; Pellino et al., 2013), the determination of potential ploidy shifts in the F_2_ plants was checked by flow cytometry. According to Carman (1997) theory, we expected that allopolyploid F_2_ hybrids would have higher frequencies of apospory and apomictic seed formation than diploid ones due to asynchrony of gene expression. We expect an increase of apospory, not only from the first hybrid generation to the next, but also higher frequencies in F_2_ plants descending from both aposporic parents, due to (epi)allelic dosage effects (Nogler, 1984b). Apomictic reproduction can be passed on to the next plant generation by male pollen (Nogler, 1984a; Van Dijk et al., 1999), which led to the assumption that maternal, aposporous plants pollinated by an aposporous paternal plant will result in an accumulation of apomictic dosage effects in the offspring. Furthermore, we carefully analyzed female development to test whether similar severe alterations and temporal irregularities during gametogenesis occur as previously observed by Hojsgaard et al. (2014). To get insights into abortion rates during seed development we analyzed seed set of the F_2_ plants. To test the hypothesis that diploid hybrid plants are also capable of producing apomictic seeds, the well-developed seeds were analyzed by flow cytometric seed screening. This step is depending on successful coupling of apospory to parthenogenesis and proper endosperm formation. Finally, by generating manual crosses of the F_2_ plants and by raising F_3_ seedlings, we have experimentally proven the viability of the next hybrid generation.

## Materials and methods

### Plant materials

Two hundred synthetic F_2_ hybrid plants were generated from crossing diploid F, J, and triploid G plants that had shown apospory before (Table S1, Hojsgaard et al., 2014). Since the F_1_ had formed almost no apomictic seed (Hojsgaard et al., 2014), the F_2_ is expected to have maternal and paternal genome contributions. All plants were grown under equal outdoor conditions in the old botanical garden of the Albrecht-von-Haller Institute for Plant Sciences at the University of Goettingen, Germany. First flowering of the plants occurred after 2–3 years of cultivation.

### Ploidy determination

The ploidy of the F_2_ plants was determined by analyzing leaf material by flow cytometry (Matzk et al., 2000; Hojsgaard et al., 2014). Small Silica gel-dried leaf pieces of ~5 mm2 were chopped in 200 μl Otto I buffer (Otto, 1990) with a razor blade and filtered through a CellTrics® filter (30 μm mesh, Sysmex Partec GmbH, Görlitz, Germany) into a flow cytometry sample tube (3.5 ml, 55 × 12 mm, Sarstedt, Nümbrecht, Germany). DNA in the filtrate was stained by adding 800 μl DAPI-containing Otto II buffer (Otto, 1990). The fluorescence intensity of stained leaf nuclei were performed with a CyFlow^®;^ Space flow cytometer (Sysmex Partec GmbH) at a gain of 416 nm. As ploidy references di- and polyploid F_1_ hybrid plants analyzed by Hojsgaard et al. (2014) were used. For all samples a minimum of 3,000 nuclei was counted and data analyses were done with the FloMax software version 2.81 (Sysmex Partec GmbH).

### Genotyping of F_2_ plants

A simple sequence repeat (SSR) genotyping approach was conducted to verify the parentage of plants. In order to exclude spontaneous selfing, unintended cross-contaminations during manual pollination as well as clonal, apomictic origin of the F_2_ *Ranunculus* generation, six loci (Table 1) were used to verify the hybrid origin of the plants following the genotyping protocol of Klatt et al. (2016). Genomic DNA was extracted from dried leaf samples using the DNeasy Plant Mini Kit (Qiagen) according to the manufacturer's protocol. Polymerase chain reactions (PCR) were performed with a final sample volume of 25 μl, containing 1 μl template DNA, 12.5 μl BIOMIX (Eurofins Genomics, Ebersberg, Germany), 0.2 μl Forward primer (10 μM), 1.0 μl Reverse primer (10 μM), 1 μl CAG-primer (FAM or HEX labeled). PCR reactions were achieved in a BioRad TM100™ Thermal Cycler with the following parameters: 94°C for 10 min, then 14 × (denaturation at 94°C for 60 s, annealing at 62°C + 0.5°C per cycle for 90 s, extension at 72°C for 60 s), followed subsequently by 35 × (denaturation at 94°C for 30 s, annealing at 55°C for 30 s and extension at 72°C for 30 s), last extension step at 72°C for 60 s and final storage conditions at 4°C. PCR sample concentrations were adjusted before 85 μl formamide (HiDi) were added. This mixture was run in an automatic capillary sequencer Genetic Analyzer 3130 (Applied Biosystems, Forster City, CA, USA) using GeneScan 500 Rox (Applied Biosystems) as size standard after a denaturing pretreatment for 3 min at 92°C. Scoring of the electropherograms was done using GeneMarker V2.4.2 (SoftGenetics LLC, State College, PA, USA) a binary presence/absence matrix of alleles was exported for genotype characterization because of the presence of several “null” alleles, which may be due to the hybrid origin of the parent plants. The SSR profiles were analyzed in FAMD applying the Jaccard similarity index and generating neighbor joining trees (Schlueter and Harris, 2006). The visualization of trees was done in FigTree v1.4.2 (Rambaut, 2009; Figures S4–S14). The data confirmed non-maternal offspring and parental combinations in the F_2_ generation (Tables S2–S12).

**Table 1:** Characteristics of the six SSR markers used for F_2_ hybrid genotyping. T_a_ (annealing temperature).

**Locus**	**Primer Sequences (5^′^-3^′^)**	**T_a_ [^°^C]**	**Repeat motif**	**References**
LH08	F: GGAGGATATGAGCGGTTCGA	54	(CA)_8_(TA)_7_	Klatt et al., 2016
	R: TATGATGCGTATGGGCGGAG	55		
LH09	F: TTATACGTGACCATCCGCCG	55	(TG)_6_(CG)_4_	Klatt et al., 2016
	R: CATTTTCAATGGTGCGAATACGA	53		
R84	F: CATCCGAAGTTAGGGTTGGA	60	(CAA)_9_	Here
	R: GAGAAAGGTGTGAGCTTGGG	60		
LH11	F: CCAACGGACACTGCTCTTCT	55	(TC)_18_	Klatt et al., 2016
	R: TGCTACTCAACCTTGAACTCGA	54		
R2562	F: TACCGCAACAACAATGAAGG	60	(TC)_22_	Here
	R: ATCTCACAAATTTGCCGTCC	60		
R2477	F: CACCTGGTTCTGGTCCTGTT	60	(TC)_16_	Here
	R: GAGCGTGTGCAACAACTCAT	60		

### Female development

To evaluate the frequency of aposporous initial cell formation in contrast to the occurrence of sexually derived functional megaspores in ovules of *R. auricomus* hybrids, differential interference contrast (DIC) microscopy was applied (Hojsgaard et al., 2014).

*Ranunculus* flower buds with a minimal diameter of 5 mm were harvested and directly fixed in FAA solution (formaldehyde: acetic acid: ethanol: dH_2_O; 2:1:10:3.5) for 48 h at room temperature. The fixative was replaced with 70% ethanol, in which samples were stored until further treatments. Thereafter, plant tissue was dehydrated using 95% and 100% ethanol for each 30 min, before the flower buds were cleared in an increasing dilution series of methyl salicylate (25; 50; 85; 100%; Carl Roth GmbH + Co. KG, Karlsruhe, Germany) in ethanol (Young et al., 1979; Hojsgaard et al., 2014). Complete ovaries were dissected from the flower buds and mounted in pure methyl salicylate on object slides. DIC microscopy analysis was performed with Leica DM5500B microscope equipped with a DFC 450 C camera and LAS V41 software (Leica Microsystems, Wetzlar, Germany).

**Figure 1:**
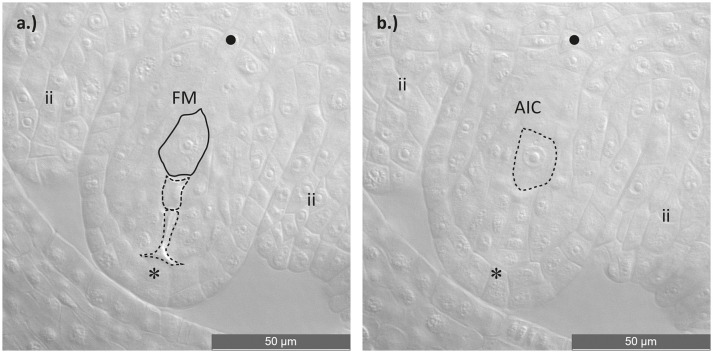
Asexual embryo sac development in an ovule of a diploid *Ranunculus* F_2_ hybrid. **(a)** Ovule during functional megaspore formation. The germ line with the four meiotic products is visible, of which three cells are aborted and only the one near the chalazal pole survived and developed into a functional megaspore. **(b)** Identical ovule as in **(a)**. but this image displays one cell layer above the germ line, showing an aposporous initial cell. Plant individual: J10 × J30 (12). FM, functional megaspore; AIC, aposporous initial cell; ii, inner integuments; *, micropylar pole; •, chalazal pole. Scale bar: 50 μm.

Discrimination of sexual and aposporic cells was accomplished by evaluation of the location of the two cell types. While sexual megaspores usually occurred at the chalazal site of the degraded germ line, asexual initial cells were found close to the sexual megaspores but obviously in somatic ovule tissue. In some ovules a temporal coexistence of functional megaspore and potential aposporous initial cell was observed (Figure 1). Percentages of sexual functional megaspores (FMs), functional aposporous initial cells (AICs) and aborted ovules are given in Table 2.

**Table 2:** Analysis of female development in diploid *Ranunculus* F_2_ hybrid ovules at the end of sporogenesis and beginning of gametogenesis.

**Hybrids**	**Type**	**No. plants**	**No. of ovules**	**FM (range)**	**Potential AIC (range)**	**Aborted ovule (range)**
F10 × F7	mp	4	159	0.45 (0.20–0.71)	0.23 (0.14–0.30)	0.32 (0.00–0.60)
J10 × J30	mp	8	710	0.68 (0.27–0.94)	0.14 (0.06–0.21)	0.18 (0.00–0.63)
J24 × J22	mp	11	360	0.72 (0.54–0.94)	0.26 (0.06–0.44)	0.02 (0.00–0.27)
F3 × J6	m	11	838	0.45 (0.11–0.85)	0.24 (0.01–0.89)	0.31 (0.00–0.64)
F7 × J9	m	3	169	0.61 (0.18–0.84)	0.14 (0.10–0.18)	0.25 (0.00–0.72)
J6 × F3	p	14	992	0.60 (0.21–0.98)	0.08 (0.00–0.19)	0.32 (0.00–0.79)
J6 × F7	p	8	427	0.78 (0.50–0.93)	0.18 (0.05–0.50)	0.04 (0.00–0.31)
J10 × J14	m	3	278	0.50 (0.25–0.67)	0.10 (0.07–0.16)	0.39 (0.16–0.68)
J20 × J2	m	14	795	0.67 (0.06–1.00)	0.09 (0.00–0.20)	0.24 (0.00–0.94)
J30 × J18	m	3	83	0.82 (0.80–0.85)	0.18 (0.15–0.20)	0.00
Total		79	4,811	63.08%	16.08%	20.85%

*Mean percentages of sexual functional megaspore (FM) formation, aposporous initial cell (AIC) formation and ovule abortion were determined by DIC microscopy. “Type” designates whether only the maternal (m) or the paternal (p) parent or both (mp) of the hybrid class was aposporous (see Table S1)*.

Statistical analyses and test for significant differences of the two groups (one parent vs. both parents aposporous) were done by applying an arcsin transformation and one-way ANOVA using IBM SPSS Statistics 24 (IBM Deutschland GmbH, Ehningen, Germany).

### Seed set

To determine the reproductive fitness of the *Ranunculus* F_2_ hybrids by seed formation, the plants were transferred from the botanical garden to a YORK® climate chamber (18°C, humidity of 60%, day: night regime of 16 h: 8 h; Johnson Controls, Milwaukee, WI, USA) to prevent unwanted pollination events e.g., by bees or other insects. At least three flowers per plant were manually cross pollinated and subsequently packed in plastic Crispac bags (2 mm Ø holes, Baumann Saatzuchtbedarf, Waldenburg, Germany) to collect ripe seeds. Harvested *Ranunculus* seeds were visually assessed and mature, brown achenes were counted and separated from aborted, yellow ones. Furthermore, full endosperm development was tested by shortly applying thumb-pressure to each achene (Klatt et al., 2016). Based on these numbers, the seed set was calculated for single collective fruits, for individual plants as well as for each hybrid cross after Hörandl (2008). Seeds were stored at 4°C until usage.

### Flow cytometric seed screen (FCSS)

The unique development pathways of single *Ranunculus* hybrid seeds were comprehended by flow cytometric measurements and data analysis (Matzk et al., 2000). Single seeds were ground by two small steel beads (4 mm Ø, Qiagen, Hilden, Germany) in a 2 ml SafeSeal micro tube (Sarstedt) using a TissueLyser II (Qiagen) for 7 s at 30 Hz s^−1^. DNA extraction started with inverting the seed powder for 30 s after adding 200 μl of Otto I buffer (Otto, 1990). Subsequent procedures such as sample filtration, nuclei staining and sample measurements were identical to the ploidy determination protocol (*incl*. gain settings). In FCSS, the ploidy of endosperm and embryo in seeds (C values) were determined by calculating means of DNA content for each peak by using the FloMax software. Based on these data the “peak index” (PI) was calculated (mean peak value of endosperm/mean peak value of embryo DNA content), which allowed, together with the peak positions, the identification of the specific reproduction pathway of every single seed (Table 3, after Klatt et al., 2016, modified).

**Table 3:** Reproductive pathways of seed development of F_3_ hybrids seeds of the *Ranunculus auricomus* complex identified by Flow Cytometric Seed Screen (FCSS).

**Reproductive pathway**		**Embryo sac (ES)**	**Embryo (Em)**	**Endosperm (End)**	**Male gametes**		**Em C + (End C)**	**PI**	**End ratio (m:p)**
					**Egg cell**	**Central cell**			
A	Sexual	Reduced	Zygotic	Fertilized	1*n*	1*n*	2 C + (3 C)	1.5	2:1
B	Paternal B_III_ hybrid	Reduced	Zygotic	Fertilized	2*n*	2*n*	3 C + (4 C)	1.3	2:2
C	Maternal B_III_ hybrid	Aposporous	Zygotic	Pseudo-gamous	1*n*	1*n*	3 C + (5 C)	1.7	4:1
D	Apomictic	Aposporous	Parthenogenetic	Autonomous Endosperm	0	0	2 C + (4 C)	2.0	4:0
E	Apomictic	Aposporous	Parthenogenetic	Pseudo-gamous	0	2*x* 2*n*	2 C + (8 C)	4.0	4:4

*Nomenclature of pathways B and C was adapted from Doležel et al. (2007). PI, peak index = endosperm/embryo ploidy; m, maternal; p, paternal*.

Earlier *R. auricomus* studies (e.g., Hojsgaard et al., 2014; Klatt et al., 2016) had revealed an eight-nucleate *Polygonum* type embryo sac, and hence a peak index of 1.5 is characteristic for sexually formed seeds. These consist of a reduced embryo sac, in which fertilization of the egg cell by one sperm nucleus results in a zygotic embryo (*n* + *n*) while the two fused reduced central cell nuclei both were fertilized by the other reduced male gamete (2*n* + *n*) (Table 3, pathway A). A classical apomictic *R. auricomus* seed is considered to exhibit peak indices of 2.0–4.0 which is due to the unreduced embryo sac nuclei, the parthenogenetic development of the embryo (2*n* + 0) and either autonomous endosperm (4*n* + 0; PI = 2.0, Table 3, pathway D) or the pseudogamous formation of the endosperm by central cell fertilization by two unreduced pollen nuclei (4*n* + 4*n*; Table 3, pathway E, peak index 4.0). We regard an interpretation of pathway D as G2 peak of the embryo as unlikely as *Ranunculus* seeds always form a rapidly growing endosperm, with endosperm peaks usually being higher than embryo peaks (while G2 peaks are always much smaller). Pathway E could also result from endosperm endopolyploidy following pathway D, but nevertheless is a case of an asexual seed. The other cases of central cell fertilization by one reduced pollen nucleus (4*n* + *n*; peak index 2.5) or by two reduced pollen nuclei (4*n* + 2*n*; peak index 3.0) as typical for established *Ranunculus* apomicts (Hojsgaard et al., 2014; Klatt et al., 2016) were not detected in our study. An intermediate case between sex and apomixis is the occurrence of so-called maternal B_III_ hybrids (Table 3, pathway C). Here, an asexually formed, unreduced egg cell is fertilized by a reduced male gamete and the endosperm is developed after fertilization of the central cell by a reduced male pollen nucleus as well. This combination results in a ploidy shift of the embryo (2*n* + *n*) and endosperm peaks (4*n* + *n*) and a unique peak index of 1.7. One single case of a paternal B_III_ hybrid was found. Here, egg cell and central cell of a reduced embryo sac were fertilized by each one unreduced pollen nucleus forming a triploid embryo (*n* + 2*n*) and tetraploid endosperm (2*n* + 2*n*; peak index = 1.3; Table 3, pathway B).

### Germination rates

In order to determine the viability of seeds formed by F_2_ plants, up to ten seeds per plant from all 13 genotypes (Table 5) were sown onto sterilized Fruhstorfer soil (type P mixed with 1/3 sand), covered with quartz gravel and incubated in a YORK® climate chamber (16°C, humidity of 60%, day: night regime of 16 h: 8 h; Johnson Controls) for 10 weeks. In spring, the pots were transferred to the old botanical garden (University of Göttingen) to ensure natural sprouting conditions. Germination was checked weekly. The final germination rates were calculated after 23 weeks.

## Results

### Ploidy determination and genotyping of F_2_ hybrids

Most of the *Ranunculus* F_2_ plants from diploid parents (F, J crosses) were found to be diploid with one exception (one new triploid plant). The individuals with a “G” in the name descended from crosses of *R. cassubicifolius* (4*x*) with *R. notabilis* (2*x*) and were previously determined as triploid. As expected from the aneuploidy of the 3*x* parent plants, the F_2_ offspring was determined as 3*x*, 4*x*, and 6*x* (Table S1).

### Female development

**Figure 2:**
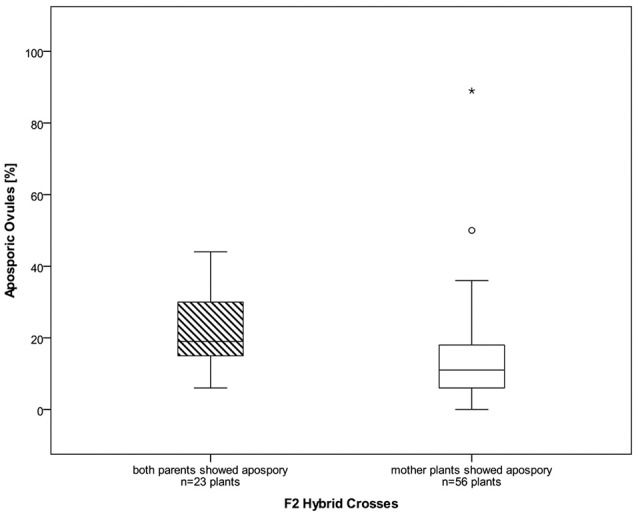
Boxplots of percentages of aposporous ovules for diploid F_2_ hybrids. Hybrid plants descending from parents that both have shown apospory before (left) formed significantly more aposporous ovules than the plants with only an aposporous mother plant (*P* = 0.012). Outliers are marked as stars and open circles, the box represents the interquartile range and in the boxplots the median is displayed.

About 4,900 ovules, from ten different synthetic *Ranunculus* F_2_ hybrid crosses, corresponding to 79 plant individuals, were examined for the mode of female development. All analyzed ovules belong exclusively to diploid *Ranunculus* plants because polyploid individuals in general formed only a very small number of flower buds. The fraction of these buds which showed the informative stage of development was too small to be statistically analyzable. The same was true for the crossings J9A × J20A, F10 × J33 and F7A × J9. Altogether, 4,811 ovules from 79 diploid plants were interpretable. Ovules showed disturbed megasporogenesis indicated by persistence of meiotic germ cell proliferation at manifold time points. The normal, sexual trait, in which the germ line cell located closest to the chalazal pole developed further into a functional megaspore, was observed in 63.08% (mean of all *Ranunculus* samples, Table 2; Figure S3a). An overall mean percentage of 16.08% of all analyzed hybrid ovules was found to develop aposporously (Figure S1; Table 2). Apospory was indicated by the occurrence of AIC in close proximity to a sexual functional megaspore, for instance one cell layer below (Figure 1). AICs occurred in two hybrid classes with an aposporous father, in five with an aposporous mother, and in three classes where both parents were aposporous (Table 2). The proportion of apospory in the analyzed hybrids derived from both aposporous parents (mean 21.18% ± 11.83 STD, median 19%) was higher in comparison to F_2_ plants that originate from parents, of which only one formed aposporic embryo sacs (mean 13.98% ± 13.94 STD, median 11%). The difference was statistically weakly significant (*P* = 0.012, Figure 2).

### Seed set, flow cytometric seed screen, germination rates

The *R. auricomus* F_2_ hybrids were used to create seed by hand-pollination between individuals in 2016. This seed set revealed a mean of 22.49% well-developed, mature *Ranunculus* seeds, while the remaining 77.51% were identified as aborted (Figure S2; Table 3). None of the polyploid F_2_ plants was able to form mature, living seeds for analysis.

**Figure 3:**
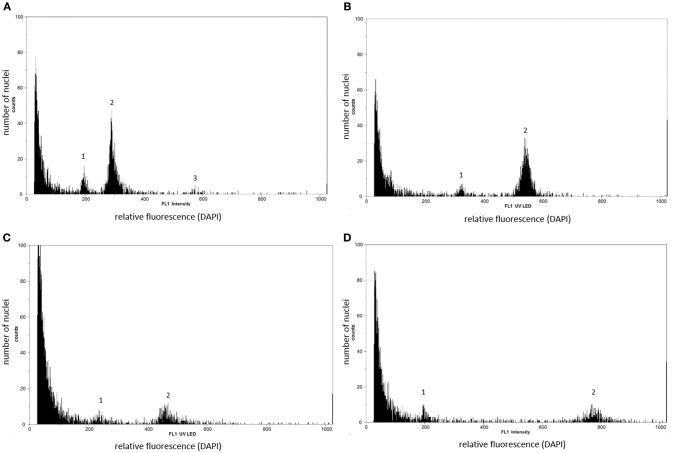
Flow cytometry histograms of *Ranunculus* F_3_ hybrid seeds formed from diploid F_2_ parents **(A–D)**. General peak labeling: 1 embryo peak, 2 endosperm peak, 3 peak of endosperm cells in the G_2_ phase of the cell cycle. **(A)** Sexual seed with a diploid embryo and triploid endosperm (pathway A). **(B)** Maternal B_III_ hybrid seed with a triploid embryo and a pentaploid endosperm tissue. The embryo sac was formed by apospory, which led to a diploid embryo and a tetraploid endosperm, but both the embryo and the central cell got fertilized by each one reduced male gamete (pathway C). **(C)** Asexual seed with a diploid embryo and a tetraploid endosperm (pathway D). The embryo, derived from an unreduced embryo sac, as well as the endosperm developed without fertilization. **(D)** Asexual *Ranunculus* seed with a diploid embryo and a near octoploid endosperm (pathway E). From the unreduced embryo sac, the embryo developed parthenogenetically into a diploid embryo and the unreduced polar nuclei got both fertilized by two unreduced pollen nuclei. Genotypes: **(A,D)** J30 × J18 (01) X J10 × J30 (04), **(B)** F10 × J33 (12) X F10 × J33 (07), **(C)** J20 × J2 (14) X J20 × J2 (18).

In the FCSS analysis only plants were taken into account that produced at least three mature, viable seeds that displayed both an embryo and an endosperm peak in FCSS histograms. Overall, 600 mature F_3_ hybrid seeds were analyzed by single-seed FCSS to elucidate their individual mode of development. The measurements showed that seven out of twelve *Ranunculus* crosses had exclusively formed sexual seeds, while the others developed B_III_ and apomictic seeds as well (Figure 3; Tables 3, 4). In total, fourteen non-sexual seeds were detected, which equals 2.33% of the 600 investigated seeds. Eleven (78.57%) of these seeds were classified as maternal B_III_ hybrids, one as paternal B_III_ hybrid and the other two apomictic seeds developed either as shown in pathway “D” or as in pathway “E” (Figure 3; Table 3).

**Table 4:** Percentage of reproductive mode found in *R. auricomus* hybrid seeds harvested from synthetic F_2_ plants.

**Hybrid parent**	**No. of seeds**	**PI (Range)**	**Reproductive pathways (Table 1)**	**Non-sexual seeds [%]**
F10 × F7	38	1.47 (1.40–1.59)	A	0.0
J10 × J30	43	1.49 (1.42–1.59)	A	0.0
J24 × J22	35	1.50 (1.43–1.69)	A	0.0
F3 × J6	111	1.48 (1.23–1.70)	A	5.13
	6	1.65 (1.65–1.66)	C	
F7A × J6	9	1.52 (1.44–1.71)	A	0.0
F7 × J9	8	1.48 (1.24–1.55)	A	0.0
F10 × J33	59	1.49 (1.41–1.64)	A	3.28
	2	1.67 (1.66–1.69)	C	
J6 × F3	107	1.48 (1.30–1.70)	A	1.82
	1	1.32	B	
	2	1.70 (1.67–1.73)	C	
J6 × F7	95	1.48 (1.33–1.58)	A	0.0
J9A × J20A	6	1.48 (1.44–1.51)	A	0.0
J10 × J14	4	1.50 (1.49–1.51)	A	0.0
J20 × J2	62	1.49 (1.38–1.61)	A	3.13
	1	1.59	C	
	1	1.93	D	
J30 × J18	9	1.46 (1.43–1.51)	A	10.00
	1	3.92	E	
Total	600	–		2.33

*Mean peak indices (PI) of the respective developmental pathways*.

In total, nearly 280 *Ranunculus* seeds were sown in February 2017 and cultivated in a climate chamber and afterwards outside in the botanical garden under natural conditions. The overall germination rate of all 13 different tested genotypes was determined after 23 weeks and was in the mean 36.96% (Table 5).

**Table 5:** Germination rate of mature *Ranunculus* seeds derived from F_2_ plants after 23 weeks of cultivation.

**Genotype (2017)**	**Maternal genotype**	**No. of seeds**	**Germination rate [%]**
F_3_-A	F10 × F7	5	60.00
F_3_-B	F10 × J33	50	36.00
F_3_-C	F3 × J6	20	30.00
F_3_-E	F6A × J15B	10	30.00
F_3_-F	F7 × J9	3	100.00
F_3_-G	F7A × J6	10	10.00
F_3_-K	J15 × F6A	10	10.00
F_3_-L	J20 × J2	36	41.67
F_3_-M	J24 × J22	36	55.56
F_3_-N	J30 × J18	5	40.00
F_3_-O	J6 × F3	72	31.94
F_3_-P	J6 × F7	9	66.67
F_3_-Q	J9A × J20A	10	10.00
Total	13	276	36.96

## Discussion

Gametophytic apomixis is a long studied topic in developmental and evolutionary botany (e.g., Winkler, 1908; Gustafsson, 1946; Nogler, 1984a). Its functional causes, however, are still unclear and under extreme debate because manifold hypothesis and ideas circulate in order to explain this phenomenon. The most important potential natural triggers are hybridization (Ernst, 1918; Mogie, 1992), polyploidization (e.g., Sober, 1984) or a combination of both (e.g., Bierzychudek, 1985; Asker and Jerling, 1992). This specific type of reproduction demands three synchronized and balanced phases to ensure growing of viable, apomictic fruits (Grimanelli et al., 2001). First, the effective circumvention of meiotic cell division (e.g., via apospory), then the parthenogenetic establishment of an embryo and finally, the successful endosperm development. In the present study synthetic *R. auricomus* hybrid plants of the second generation were analyzed. To common knowledge sexual *Ranunculus* plants follow the *Polygonum* type of female development (Nogler, 1973) but evidently this important process was heavily altered, indicated by persistence or abortion of embryo sac formation. Similar but more severe developmental disturbances have been described by Hojsgaard et al. (2014), who analyzed the parental generation of the plants in focus here.

### Frequencies and genomic dosage effects on apospory

All analyzed *Ranunculus* plants of the second hybrid generation were invariably identified as diploid, non-maternal genotypes, which means that these plants were sexually formed without any spontaneous ploidy shift. In the grand mean, 16.08% of the investigated F_2_ ovules showed aposporous initial cell formation, while only a mean of 11% of the diploid F_1_ hybrid ovules had aposporous development (Hojsgaard et al., 2014). In addition, apospory in F_2_ hybrids seems to be dependent on dosage of heritable genetic control factors, because plants that originated from parents that both had shown apomeiotic embryo sac development, displayed a significantly enhanced percentage of asexual ovules (21.18% ± 11.83 STD) compared to individuals with only one aposporous parent (13.98% ± 13.94 STD, *P* = 0.012). Since all plants were kept under equal conditions, we can rule out differential stress influence on frequencies of apospory (Klatt et al., 2016; Rodrigo et al., 2017). The influence of genomic dosage of control factors on frequencies of aposporous ovule formation was already shown previously in crossing experiments of polyploid *Ranunculus* by Nogler (1984b). However, other than assumed by Nogler (1984a), our results suggest that also haploid male and female gametes can carry apospory-controlling heritable control factors. *Ranunculus* F_2_ hybrids illustrate developmental disturbances during megasporogenesis and -gametogenesis that are often thought to result in either whole ovule abortion or in reduced fertility due to failures during megagametophyte formation (Figures S3b–d). Similar, but more drastic irregularities were observed by Hojsgaard et al. (2014) when analyzing the temporal and developmental processes during female development of the F_1_ generation. In contrast, natural *Ranunculus* hybrids show milder discrepancies in embryo sac and seed formation (Nogler, 1971, 1972; Hojsgaard et al., 2014).

However, it is still unresolved which apomeiosis-provoking factor triggers reprogramming of a somatic nucellar cell. Since an effect of polyploidy can be ruled out in our F_2_ plants, it is assumed that all these alterations are due to previous hybridization, which consequences are known to be the strongest and most perceptible in the first few hybrid generations, especially in diploid plants (Barton, 2001). Hybridization is a powerful driving force in plant speciation and evolution that can result in genomic shocks (Rieseberg et al., 2003). Hybridization can cause dramatic chromosomal rearrangements which were shown to be associated to apomixis in diploid, diplosporous *Boechera* (Kantama et al., 2007). It is further supposed that hybridization events dislocate timing and pattern of gene expression of sexual reproduction controlling genes by changing their genomic constitution or epigenetic regulation (Koltunow, 1993; Carman, 1997; Hand and Koltunow, 2014; Shah et al., 2016). Epigenetics is altered upon hybridization in plants and such reversible changes like DNA methylation or RNA interference are thought to be able to cause apomixis, without affecting the plants' genome sequence (Comai, 2005; Ozias-Akins and van Dijk, 2007; Grimanelli, 2012). In Ha et al. (2009) speculated that genomic shocks can be prevented by specific small RNAs formed during hybridization or polyploidization, providing improved genome stability to hybrid plants. Furthermore, it was shown that the onset of reproductive actions in mutant *Arabidopsis* ovules were mainly caused by small RNA silencing pathways involving the AGO9 protein (Olmedo-Monfil et al., 2010). Cell-to-cell signaling is a feature of double-stranded small RNAs, where they tend to silence their target genes (Molnar et al., 2010). The assumption of cell-to-cell signaling is reasonable because aposporous initials always emerge in the direct neighborhood of the megaspore tetrad (e.g., Figure 1). Small RNAs commonly interact with proteins of the ARGONAUTE family and form together the RNA-induced silencing complex (RISC), which is an essential component during transcriptional and posttranscriptional gene silencing (Bourc'his and Voinnet, 2010; Feng et al., 2010; Mallory and Vaucheret, 2010). Thus, it seems reasonable that heritable epigenetic processes are responsible for functional silencing of the sexual reproduction pathway in favor of apomixis (Grimanelli, 2012; Hand and Koltunow, 2014).

Our results confirm that apospory is a facultative mechanism, which includes parallel existence of sexual and apomictic development and thus finally in a mixture of sexual and asexual seeds (Nogler, 1984a). The facultative character of gametophytic apomixis could be the result of the ability to maintain the epigenetically unsilenced genomic state (Hand and Koltunow, 2014).

### Diploid hybrids are able to reproduce via apomictic seed formation

During seed formation, further developmental processes come into play and influence proportions of sexually vs. apomictically formed seed. Successful seed formation in sexual *Ranunculus* species is highly dependent on fertilization of the egg and the central cell nuclei. In pseudogamous apomicts, fertilization of the central cell and endosperm development is important for seed formation as well. In angiosperms, the optimal ratio of maternal (m) to paternal (p) genome contributions in the endosperm was determined to be 2:1 due to genomic imprinting. Deviations from this ratio have deleterious effects leading to heavy disturbances or even to seed abortions (Spielman et al., 2003; Vinkenoog et al., 2003). *Ranunculus* species were characterized as very sensitive to endosperm imbalances (Hörandl and Temsch, 2009). Failure of endosperm development likely explains high seed abortion rates of our F_2_ hybrids. More than three-fourth of all seeds harvested from diploid *Ranunculus* F_2_ hybrids were found to be dead, either due to abortion at early stages of development or due to mal-developed endosperm tissue (Figure S2). Only a mean of 22.49% of achenes were intact. Conversely, polyploid F_2_ hybrids failed completely to produce mature seeds. Even well-formed achenes showed no embryo peak in FCSS analyses. Therefore, no evaluation on the reproductive mode from polyploid plants could be made. Extreme seed abortion rates in the diploids appeared to be at expense of apomictic development as finally almost only functional sexual seeds were formed. This differs fundamentally from natural *Ranunculus* apomicts that produce higher proportions of apomictic than sexual seeds (Hojsgaard et al., 2014; Klatt et al., 2016).

In other apomictic plant genera the assertiveness of the sexual pathway seem to mainly depend on the survival rate of functional meiotic cells that possibly can be influenced by pollination timing (Espinoza et al., 2002; Hojsgaard et al., 2013). Natural apomicts have found various strategies to circumvent seed failure e.g., by sustaining the optimal conditions or by tolerating variations in the paternal contribution to the endosperm (e.g., Savidan, 2007; Dobeš et al., 2013). In our synthetic F_2_ plants, seed formation trod various paths. Most of the diploid plants developed sexual seeds and maintained the favored conditions of 2m: 1p ratio, while the apomictic seeds showed several types of genomic imbalance based on an unreduced embryo sac. B_III_ hybrid seeds revealed a different endosperm contribution of 4m: 1p or 2m: 2p (Table 3). The seed that developed fully autonomously without any fertilization showed an extreme change to 4m: 0p (Table 3) and the seed that followed reproductive pathway D showed a modified genomic imbalance in the endosperm of 4m: 4p (Table 3). A comparable tolerance was also previously observed in polyploid F_1_ plants, which showed alterations in genome dosage as well (Hojsgaard et al., 2014). Autonomous endosperm development was before reported for apomictic *R. auricomus* (Klatt et al., 2016), and here analysis of the FCSS histogram verifies this rare observation (Figure 3C). Also in apomictic *R. kuepferi*, autonomous endosperm occurred very rarely (Schinkel et al., 2016).

However, the two most common modes of seed formation in natural apomictic *Ranunculus* were not detected in this study. Typical apomictic *Ranunculus* seeds are composed of an unreduced embryo sac that parthenogenetically developed into an embryo plus a pseudogamously developed endosperm formed by fertilization of one unreduced or two reduced male gametes (PI = 2.5 and 3.0 respectively, e.g., Klatt et al., 2016). Notably, these cases restore the optimal 2m: 1p ratio in the endosperm and usually represent the most frequent case of functional apomictic seeds in *Ranunculus* (Hojsgaard et al., 2014). It is likely that none of these “standard” cases was found in the F_2_ hybrids due to various reasons: (1) in many cases, premature embryo sac formation during the bud stage was observed, before pollen is available (Figures S3e,f); (2) the pollen quality of hybrid plants could have been low, as it is typical for *Ranunculus* apomicts (Izmailow, 1996; Hörandl et al., 1997; Schinkel et al., 2017); in both cases, failure of endosperm development could have caused seed abortion. Alternatively, several crosses (F × F and J × J genotypes) could have resulted in sibling cross incompatibility.

The formation of several B_III_ hybrids by diploid F_2_ hybrids indicates an insufficient coupling of apospory and parthenogenesis, which are both essential for the functional establishment of gametophytic apomixis. This circumstance could be due to the early onset of apospory in F_1_ hybrids as described by Hojsgaard et al. (2014) and again verified in the F_2_ plants by appearance of fully mature, seven-nucleic embryo sacs already in flower bud stage (Figures S3e,f). It is assumed that apospory is connected to extreme long time periods of egg cell receptivity, which reduces the degree of parthenogenetically formed embryos and in turn increases the number of B_III_ seeds as found in the F_2_ hybrids (Martinez et al., 1994; Nogler, 1995). Apospory and parthenogenesis are under different genetic control mechanisms (Nogler, 1984a; Ozias-Akins and van Dijk, 2007). The coupling of these processes is obviously not yet established in the majority of F_2_ hybrids studied here, except for two apomictically formed seeds.

### The role of polyploidy for expression of apomixis

Our results shed a new light on the role of polyploidy. In almost all plants, natural apomictic reproduction occurs together with polyploidization, which led to the conclusion that polyploidy is an essential necessity for apomixis rather than an option (e.g., Bierzychudek, 1985; Carman, 1997; Koltunow and Grossniklaus, 2003). Nonetheless, a few reports on natural diploid apomictic plants are known e.g., in *Boechera* (Dobeš et al., 2006; Aliyu et al., 2010), *Paspalum* (Siena et al., 2008), and *R. kuepferi* (Schinkel et al., 2016). Thus, the switch to gametophytic apomixis in the F_2_ generation analyzed here is maintained by the hybrid character of the plants and not by polyploidization. Nevertheless, the identified B_III_ hybrid seeds, derived from diploid parents, would result in triploid neopolyploids with a high potential for apospory because of maternal gene dosage effects. Fertilization of the unreduced triploid egg cell by an aposporous pollen donor will result in tetraploids with increased dosages for apospory. So-called female triploid bridges are described in mediating polyploid apomicts (e.g., Schinkel et al., 2017). In this aspect our results support the hypothesis by Schinkel et al. (2017) that apomixis would be rather a cause than a consequence of polyploidy.

Flow cytometric observations of apomictically developed diploid *Ranunculus* seeds do not support the hypothesis by Nogler (1984a) that inheritance of apospory-controlling factors would require unreduced gametes because of recessive lethal effects in the haploid genome. Mature diploid, asexual *Ranunculus* seeds show that they do not suffer from recessive lethal effects during seed formation. Since hybridization events are known to cause disturbances in epigenetic regulation, the observed irregularities and temporal alterations are likely to be due to changes of epigenetic genome modulation (e.g., Grimanelli, 2012). In order to get a complete picture of the establishment of apomixis in *Ranunculus* hybrid plants, the viability of harvested seeds, derived from synthetic F_2_ hybrids, was analyzed by determination of their germination rate. Sexual *Ranunculus* species are known to have a higher fitness than apomictic species in terms of seed set (Izmailow, 1996; Lohwasser, 2001; Hörandl, 2008). However, germination rates between diploid F_1_ hybrids, hexaploid apomicts, and diploid sexual species did not differ significantly from each other (Hörandl, 2008). The observed mean germination rate of c. 37% in the F_2_ hybrids studied here falls within the range of means of c. 35–54% of the previous study. The germination process is obviously not significantly disturbed in F_2_ hybrids, which means that further hybrid generations can be formed.

## Conclusion

The success of apospory in diploid *Ranunculus* F_2_ hybrids was found to be based on irregularities during female development, triggered by interspecific hybridization that strongly interfered with temporal and developmental course of action. The frequency of unreduced embryo sac formation depended on the dosage of genetic control factors passed on by the parent generation. However, the connection of apospory, parthenogenesis and pseudogamous endosperm formation is not yet reliably installed in synthetic F_2_ hybrids, which is indicated by a high rate of aborted seeds, probably suffering from unbalanced maternal: paternal genome contributions to the endosperm. Nevertheless, a small but not negligible number of apomictic and B_III_ seeds was obtained, meaning that the establishment of apomixis in *Ranunculus* hybrids potentially can continue in further generations. On the one hand, B_III_ hybrid plants are assumed to be the next step toward stabilization and extension of apomictic potential in a polyploid background. On the other hand, polyploid F_2_ hybrids in this study, only formed a small number of mature but not analyzable seeds without detectable embryo tissue. Thus, for the next plant generation several triploid individuals are expected that would be highly aposporous but mostly seed-sterile. In a larger, evolutionary timescale, rare successful polyploid apomictic seed formation would be favored by natural selection and increase in frequency. This process could result in the establishment of a functional apomictic, polyploid new *Ranunculus* lineage.

## Author contributions

BB performed research, analyzed and interpreted data. BB and EH wrote the manuscript. EH designed the research. MD performed some FCSS experiments.

### Conflict of interest statement

The authors declare that the research was conducted in the absence of any commercial or financial relationships that could be construed as a potential conflict of interest.

## Abbreviations

AIC: aposporous initial cell
DIC: differential interference contrast microscopy
ES: embryo sac
FCSS: flow cytometric seed screen
FM: functional megaspore
SSR: simple sequence repeat.
