# Supplementary material for: Establishment of Apomixis in Diploid F2 Hybrids and Inheritance of Apospory From F1 to F2 Hybrids of the Ranunculus auricomus Complex
Source: Front Plant Sci. 2018 Aug 3;9:1111. doi: 10.3389/fpls.2018.01111 (PMC6085428; doi:10.3389/fpls.2018.01111)
Supplement: Supplementary file 1 [file Image_1.PDF]

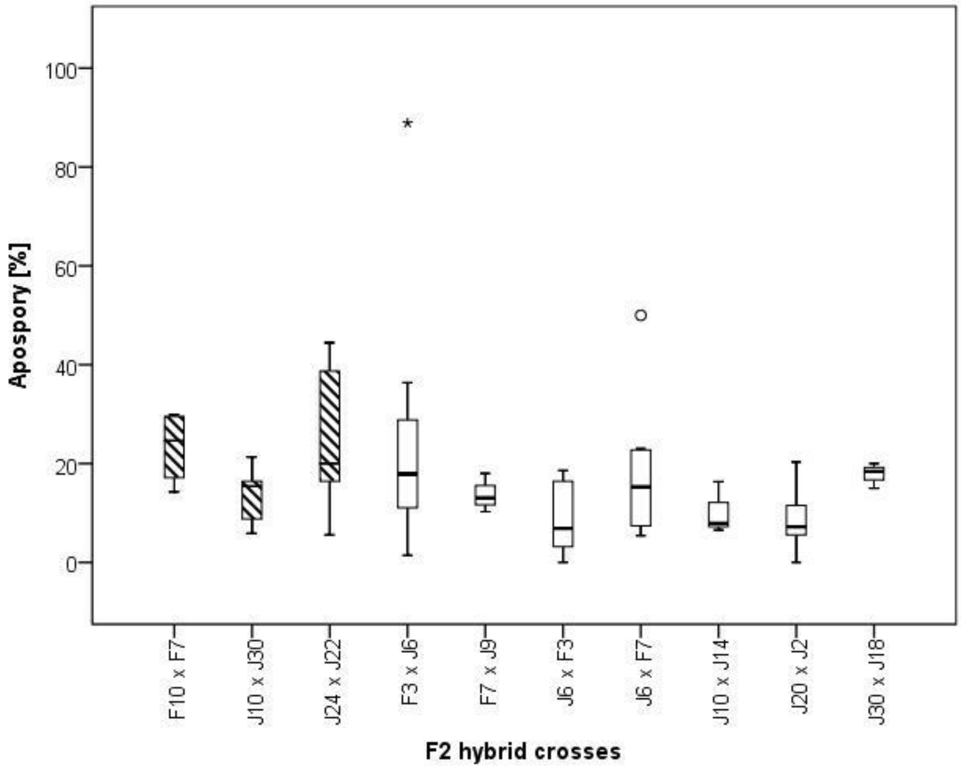

Figure S1: Mean percentage of apospory found in ovules of Ranunculus F_2 hybrids. The percentage of apospory varies between 7 and 25 % in Ranunculus F_2 hybrid ovules. Striped box plots correspond to F_2 crosses that descend from parents, which both have shown apospory before. Hybrid crosses depicted as white box plots have only an aposporous mother or father plant. N – Numbers are listed in Table 3. Outliers are marked as stars and open circles and in the boxplots the median is displayed.
