# Supplementary material for: Establishment of Apomixis in Diploid F2 Hybrids and Inheritance of Apospory From F1 to F2 Hybrids of the Ranunculus auricomus Complex
Source: Front Plant Sci. 2018 Aug 3;9:1111. doi: 10.3389/fpls.2018.01111 (PMC6085428; doi:10.3389/fpls.2018.01111)
Supplement: Supplementary file 2 [file Image_2.pdf]

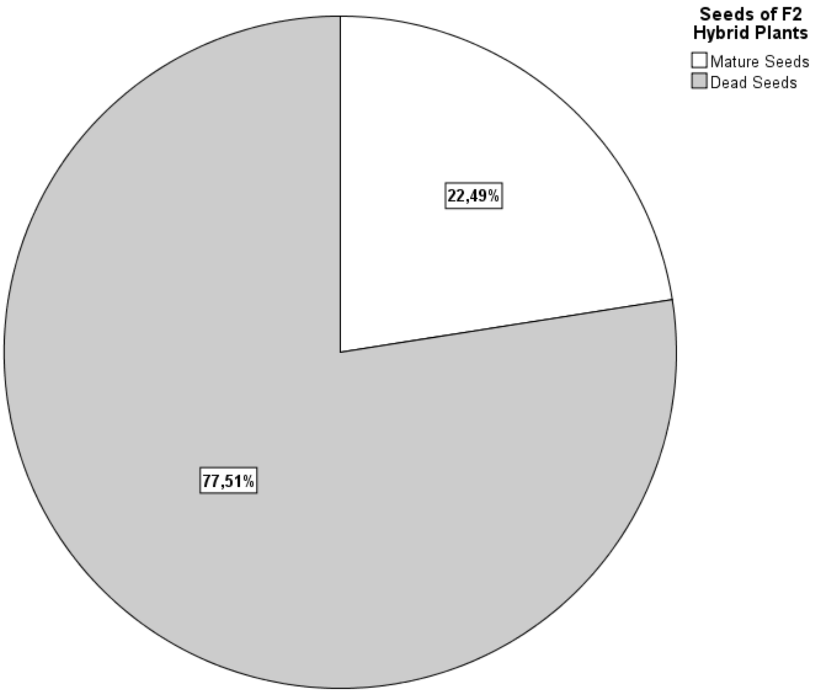

Figure S2: Mean seed-set of diploid *Ranunculus auricomus* hybrids. The F_3 seeds were produced by hand-pollination of the synthetic F_2 hybrids and visually and mechanically analyzed for proper development (n=8681). Turning out that only 22.49 % of the harvested seeds were mature and alive, while the rest was maldeveloped and aborted (77.51 %).
