# Supplementary material for: Establishment of Apomixis in Diploid F2 Hybrids and Inheritance of Apospory From F1 to F2 Hybrids of the Ranunculus auricomus Complex
Source: Front Plant Sci. 2018 Aug 3;9:1111. doi: 10.3389/fpls.2018.01111 (PMC6085428; doi:10.3389/fpls.2018.01111)
Supplement: Supplementary file 3 [file Image_3.pdf]

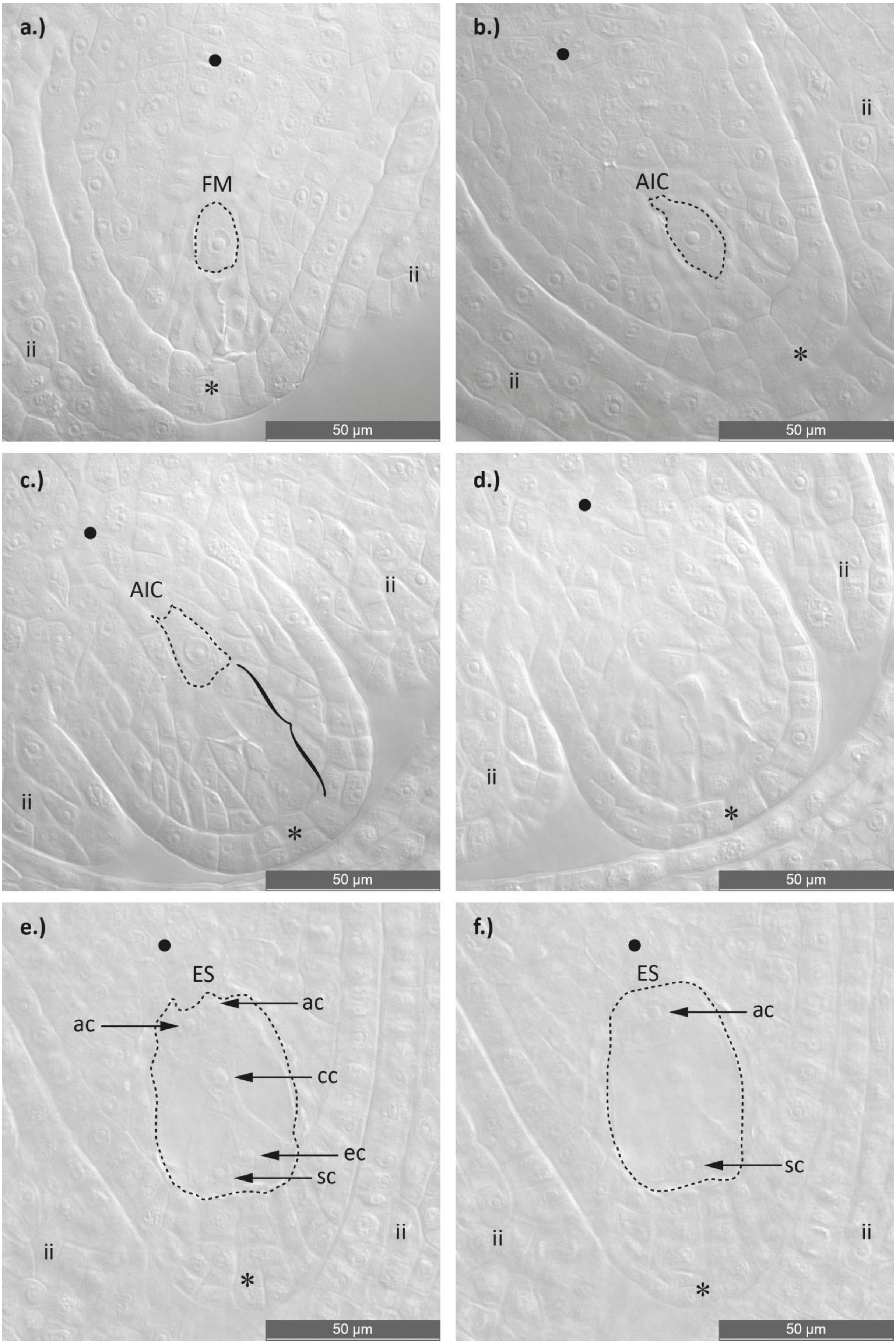
a.)
FM
ii
ii
*
50 µm
b.)
AIC
ii
ii
*
50 µm
c.)
AIC
ii
ii
*
50 µm
d.)
ii
ii
*
50 µm
e.)
ES
ac
ac
cc
ec
sc
ii
ii
*
50 µm
f.)
ES
ac
sc
ii
ii
*
50 µm

Figure S3: Different stages of ovule development in diploid *Ranunculus* F_2 hybrids in flower buds. a.) Regular, sexual ovule showing a functional megaspore after meiosis completion. b.) Aposporous initial cell, indicated by its position in the ovule and missing functional megaspore as well as aborted megaspores. c.) Aposporous initial cell, all four meiotic products are aborted. d.) Completely aborted germ line without AIC formation. e.+ f.) Precocious ovule development, mature, seven nucleic embryo sac at flower bud stage. Both figures show the same embryo sac at different cell layers. Plant individuals: a.+ b.) J10xJ30 (05); c.)+ d.) F3xJ6 (24); e.+ f.) J10xJ30 (04). FM, functional megaspore; AIC, aposporous initial cell; ES, embryo sac; ac, antipodal cell; cc, central cell both polar nuclei are already fused; ec, egg cell; ii, inner integuments; sc, synergid cell; *, micropylar pole; ●, chalazal pole; }, four aborted megaspores. Scale bar: 50 µm.
