# Supplementary material for: Establishment of Apomixis in Diploid F2 Hybrids and Inheritance of Apospory From F1 to F2 Hybrids of the Ranunculus auricomus Complex
Source: Front Plant Sci. 2018 Aug 3;9:1111. doi: 10.3389/fpls.2018.01111 (PMC6085428; doi:10.3389/fpls.2018.01111)
Supplement: Supplementary file 12 [file Image_12.pdf]

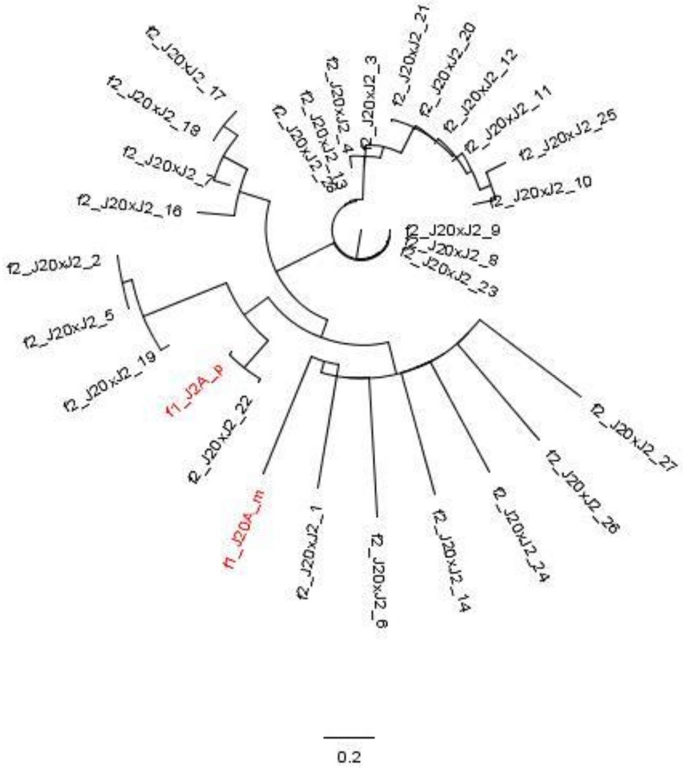

Figure S12: Neighbor joining trees derived from SSR data (all loci and alleles). Each two synthetic *Ranunculus* F_1 hybrids and their sexually formed offspring were analyzed. F_1 parent plants are depicted in red.
