# Supplementary material for: Establishment of Apomixis in Diploid F2 Hybrids and Inheritance of Apospory From F1 to F2 Hybrids of the Ranunculus auricomus Complex
Source: Front Plant Sci. 2018 Aug 3;9:1111. doi: 10.3389/fpls.2018.01111 (PMC6085428; doi:10.3389/fpls.2018.01111)
Supplement: Supplementary file 15 [file Table_1.docx]

Table S1: Natural Ranunculus auricomus species and their synthetic hybrid offspring. The F_1_ and the F_2_ generation were generated by manual crossing of the relative parent generation in 2006 and 2010, respectively. The letters F and J function as abbreviations for plants descending from R. carpaticola x R. notabilis crosses and the letters I and G belong to hybrids originating from R. cassubicifolius x R. notabilis crosses. # describes the quantity of plant individuals of a genotype and ‘ indicates a ploidy shift: J24 x J22 (21) is triploid.

| **Parental Generation**  **(Natural Plants)** | | **F_1_ Hybrid Generation (2006)** | | | **F_2_ Hybrid Generation (2010)** | |
| --- | --- | --- | --- | --- | --- | --- |
|  |  |  | **Sexual Plants** | **Apo. Plants** | **Crosses of apo. mat. Plants (No.)** | **Ploidy of F_2_ Plants** |
| *Ranunculus carpaticola* (Soó) | 2x sexual (allogamous) | *R. carpaticola* x  *R. notabilis*  (≈ J, F plants) | J2,  J6,  J9,  J14, J15,  J18, J18A, J20A, J33 | F3,  F7,  F7A,  F10,  J9A,  J10,  J20,  J22,  J24,  J30,  J30A | F3 x J6 (#31),  F7 x J9 (#4),  F7A x J6 (#4),  F10 x J33 (#19),  J9A x J20A (#1),  J10 x J14 (#20),  J20 x J2 (#28),  J30 x J18 (#4),  J30A x J18A (#3),  G12 x G7A (#1),  G16A x GI2A (#1) | 2x  2x  2x  2x  2x  2x  2x  2x  2x  6x  4x |
| *Ranunculus notabilis*  (Hörandl & Gutermann) | 2x sexual (allogamous) | *R. cassubicifolius* x *R. notabilis*  (≈ G, I plants) | G1, G7A,  I2,  I2A | G9,  G12,  G16A,  G19 | **Crosses of apo. pat. Plants (No.)** | **Ploidy of F_2_ Plants** |
|  |  |  |  |  | J6 x F3 (#37),  J6 x F7 (#16),  G1 x G9 (#2) | 2x  2x  3x, 4x |
|  |  |  |  |  | **Crosses of apo. mat. & pat. Plants (No.)** | **Ploidy of F_2_ Plants** |
| *Ranunculus cassubicifolius* (W. Koch) | 4x sexual (allogamous) |  |  |  | F10 x F7 (#6),  J10 x J30 (#14),  J24 x J22’ (#29),  G19 x G9 (#1) | 2x  2x  2x, 3x  3x |
