# Supplementary material for: Establishment of Apomixis in Diploid F2 Hybrids and Inheritance of Apospory From F1 to F2 Hybrids of the Ranunculus auricomus Complex
Source: Front Plant Sci. 2018 Aug 3;9:1111. doi: 10.3389/fpls.2018.01111 (PMC6085428; doi:10.3389/fpls.2018.01111)
Supplement: Supplementary file 18 [file Table_4.DOCX]

Table S4: Selected SSR data verifying the non-clonal origin of synthetic Ranunculus F_2_ hybrids by depicting the presence of paternal private alleles. m, maternal; p, paternal; N, drop out. The total matrix comprises six loci with altogether 33 alleles (coded as binary presence/absence data).

|  | **LH09_206** | **LH08_176** | **R84_162** | **LH11_254** | **R2562_367** | **R2562_405** | **R2477_265** | **R2477_291** |
| --- | --- | --- | --- | --- | --- | --- | --- | --- |
| **f1_F3A_m** | 0 | 0 | 0 | 0 | 0 | 0 | 0 | 0 |
| **f1_J6_p** | 1 | 1 | 1 | 1 | 1 | 1 | 1 | 1 |
| f2_F3xJ6_1 | 0 | 0 | 0 | 0 | 1 | 0 | 0 | 0 |
| f2_F3xJ6_10 | 1 | 1 | 1 | 0 | 1 | 0 | 1 | 1 |
| f2_F3xJ6_11 | 1 | 1 | 1 | 1 | 0 | 0 | N | N |
| f2_F3xJ6_12 | 0 | N | 1 | 0 | 0 | 1 | 1 | 1 |
| f2_F3xJ6_13 | 1 | 1 | 0 | 1 | 0 | 0 | 0 | 0 |
| f2_F3xJ6_14 | 1 | 1 | 1 | 0 | 0 | 1 | 1 | 1 |
| f2_F3xJ6_15 | 0 | 1 | 0 | 1 | 0 | 1 | 0 | 0 |
| f2_F3xJ6_16 | 1 | 0 | 1 | 0 | N | N | 1 | 0 |
| f2_F3xJ6_17 | 0 | 1 | 0 | 1 | 0 | 1 | N | N |
| f2_F3xJ6_18 | 0 | 0 | 0 | 1 | 0 | 0 | 0 | 1 |
| f2_F3xJ6_19 | 0 | N | 0 | 1 | 0 | 1 | 0 | 0 |
| f2_F3xJ6_2 | 0 | N | 0 | 1 | 0 | 1 | N | N |
| f2_F3xJ6_20 | 0 | 0 | 1 | 0 | 0 | 0 | 1 | 1 |
| f2_F3xJ6_21 | 1 | 0 | 0 | 1 | 1 | 0 | 0 | 0 |
| f2_F3xJ6_22 | 1 | 1 | 1 | 1 | 1 | 0 | 0 | 1 |
| f2_F3xJ6_23 | 1 | 1 | 0 | 1 | 0 | 0 | N | N |
| f2_F3xJ6_24 | 0 | N | 1 | 0 | 1 | 1 | 1 | 0 |
| f2_F3xJ6_25 | 0 | 1 | 0 | 1 | 1 | 0 | 1 | 0 |
| f2_F3xJ6_26 | 1 | 0 | 0 | 0 | 0 | 0 | 1 | 0 |
| f2_F3xJ6_27 | 1 | 1 | 1 | 0 | 0 | 0 | 1 | 1 |
| f2_F3xJ6_28 | 0 | 1 | 0 | 1 | N | N | N | N |
| f2_F3xJ6_29 | 0 | 1 | 1 | 0 | 0 | 1 | 1 | 0 |
| f2_F3xJ6_3 | 0 | 0 | 1 | 0 | 1 | 1 | 1 | 1 |
| f2_F3xJ6_30 | 0 | 0 | 0 | 1 | 1 | 0 | N | N |
| f2_F3xJ6_31 | 0 | 1 | 0 | 1 | 0 | 1 | 0 | 0 |
| f2_F3xJ6_4 | 0 | 0 | 1 | 0 | 0 | 0 | 1 | 0 |
| f2_F3xJ6_5 | 1 | N | 0 | 1 | 0 | 0 | 0 | 0 |
| f2_F3xJ6_6 | 0 | 1 | 1 | 1 | 0 | 1 | N | N |
| f2_F3xJ6_7 | 0 | 0 | 0 | 1 | 0 | 1 | 0 | 0 |
| f2_F3xJ6_9 | 0 | 0 | 1 | 1 | 0 | 0 | N | N |
