# Supplementary material for: Establishment of Apomixis in Diploid F2 Hybrids and Inheritance of Apospory From F1 to F2 Hybrids of the Ranunculus auricomus Complex
Source: Front Plant Sci. 2018 Aug 3;9:1111. doi: 10.3389/fpls.2018.01111 (PMC6085428; doi:10.3389/fpls.2018.01111)
Supplement: Supplementary file 20 [file Table_6.DOCX]

Table S6: Selected SSR data verifying the non-clonal origin of synthetic Ranunculus F_2_ hybrids by depicting the presence of paternal private alleles. m, maternal; p, paternal; N, drop out. The total matrix comprises six loci with altogether 33 alleles (coded as binary presence/absence data).

|  | **LH09_206** | **LH11_218** | **R2562_367** | **R2562_385** | **R2477_285** |
| --- | --- | --- | --- | --- | --- |
| **f1_J10A_m** | 0 | 0 | 0 | 0 | 0 |
| **f1_J14A_p** | 1 | 1 | 1 | 1 | 1 |
| f2_J10xJ14_1 | 1 | 1 | 1 | 0 | 0 |
| f2_J10xJ14_10 | 0 | 0 | 0 | 1 | N |
| f2_J10xJ14_11 | 0 | 1 | 1 | 0 | 1 |
| f2_J10xJ14_12 | 0 | 0 | 1 | 0 | 0 |
| f2_J10xJ14_13 | 0 | 0 | 1 | 0 | 1 |
| f2_J10xJ14_14 | 0 | 1 | 0 | 0 | 0 |
| f2_J10xJ14_15 | 1 | 0 | 1 | 0 | 0 |
| f2_J10xJ14_16 | 1 | 1 | 0 | 1 | 1 |
| f2_J10xJ14_17 | 0 | 1 | 0 | 0 | 0 |
| f2_J10xJ14_18 | 0 | 0 | 1 | 0 | 0 |
| f2_J10xJ14_2 | 1 | 1 | 1 | 0 | 0 |
| f2_J10xJ14_3 | 0 | 0 | N | N | N |
| f2_J10xJ14_4 | N | 0 | N | N | N |
| f2_J10xJ14_5 | 0 | 0 | 1 | 0 | N |
| f2_J10xJ14_6 | 1 | 0 | N | N | N |
| f2_J10xJ14_7 | 1 | 1 | 1 | 0 | N |
| f2_J10xJ14_8 | 1 | 0 | N | N | 0 |
| f2_J10xJ14_9 | 1 | 0 | 1 | 0 | 0 |
