# Supplementary material for: Establishment of Apomixis in Diploid F2 Hybrids and Inheritance of Apospory From F1 to F2 Hybrids of the Ranunculus auricomus Complex
Source: Front Plant Sci. 2018 Aug 3;9:1111. doi: 10.3389/fpls.2018.01111 (PMC6085428; doi:10.3389/fpls.2018.01111)
Supplement: Supplementary file 21 [file Table_7.DOCX]

Table S7: Selected SSR data verifying the non-clonal origin of synthetic Ranunculus F_2_ hybrids by depicting the presence of paternal private alleles. m, maternal; p, paternal; N, drop out. The total matrix comprises six loci with altogether 33 alleles (coded as binary presence/absence data).

|  | **LH08_162** | **LH11_218** | **R2562_385** | **R2477_285** |
| --- | --- | --- | --- | --- |
| **f1_J10A_m** | 0 | 0 | 0 | 0 |
| **f1_J30A_p** | 1 | 1 | 1 | 1 |
| f2_J10xJ30_10 | N | N | 0 | 1 |
| f2_J10xJ30_11 | N | N | 0 | 1 |
| f2_J10xJ30_12 | N | N | 0 | 1 |
| f2_J10xJ30_13 | N | N | 0 | 1 |
| f2_J10xJ30_14 | N | N | 0 | 1 |
| f2_J10xJ30_3 | 1 | 0 | 0 | 0 |
| f2_J10xJ30_4 | 0 | 0 | 0 | 0 |
| f2_J10xJ30_5 | N | N | 0 | 1 |
| f2_J10xJ30_6 | N | N | 0 | 0 |
| f2_J10xJ30_7 | N | N | 1 | 1 |
