# Supplementary material for: Establishment of Apomixis in Diploid F2 Hybrids and Inheritance of Apospory From F1 to F2 Hybrids of the Ranunculus auricomus Complex
Source: Front Plant Sci. 2018 Aug 3;9:1111. doi: 10.3389/fpls.2018.01111 (PMC6085428; doi:10.3389/fpls.2018.01111)
Supplement: Supplementary file 22 [file Table_8.DOCX]

Table S8: Selected SSR data verifying the non-clonal origin of synthetic Ranunculus F_2_ hybrids by depicting the presence of paternal private alleles. m, maternal; p, paternal; N, drop out. The total matrix comprises six loci with altogether 33 alleles (coded as binary presence/absence data).

|  | **LH11_218** | **LH11_242** | **R2562_367** | **R2562_405** |
| --- | --- | --- | --- | --- |
| **f1_J20A_m** | 0 | 0 | 0 | 0 |
| **f1_J2A_p** | 1 | 1 | 1 | 1 |
| f2_J20xJ2_1 | N | N | N | N |
| f2_J20xJ2_10 | 0 | 1 | 1 | 0 |
| f2_J20xJ2_11 | 0 | 1 | 1 | 0 |
| f2_J20xJ2_12 | 0 | 1 | 1 | 0 |
| f2_J20xJ2_13 | 1 | 0 | 1 | 0 |
| f2_J20xJ2_14 | N | N | N | N |
| f2_J20xJ2_16 | 1 | 0 | 0 | 1 |
| f2_J20xJ2_17 | 1 | 0 | 0 | 0 |
| f2_J20xJ2_18 | 1 | 0 | 0 | 0 |
| f2_J20xJ2_19 | 0 | 1 | 0 | 1 |
| f2_J20xJ2_2 | N | N | 0 | 1 |
| f2_J20xJ2_20 | 0 | 1 | 1 | 0 |
| f2_J20xJ2_21 | 0 | 1 | 1 | 0 |
| f2_J20xJ2_22 | N | N | 1 | 1 |
| f2_J20xJ2_23 | 1 | 0 | 1 | 0 |
| f2_J20xJ2_24 | N | N | N | N |
| f2_J20xJ2_25 | 0 | 1 | N | N |
| f2_J20xJ2_26 | N | N | N | N |
| f2_J20xJ2_27 | N | N | N | N |
| f2_J20xJ2_28 | 1 | 0 | 1 | 0 |
| f2_J20xJ2_3 | N | N | 1 | 0 |
| f2_J20xJ2_4 | N | N | 1 | 0 |
| f2_J20xJ2_5 | N | N | 0 | 1 |
| f2_J20xJ2_6 | N | N | N | N |
| f2_J20xJ2_7 | 1 | 0 | N | N |
| f2_J20xJ2_8 | 1 | 0 | 1 | 0 |
| f2_J20xJ2_9 | 1 | 0 | 1 | 0 |
