# Supplementary material for: Establishment of Apomixis in Diploid F2 Hybrids and Inheritance of Apospory From F1 to F2 Hybrids of the Ranunculus auricomus Complex
Source: Front Plant Sci. 2018 Aug 3;9:1111. doi: 10.3389/fpls.2018.01111 (PMC6085428; doi:10.3389/fpls.2018.01111)
Supplement: Supplementary file 23 [file Table_9.DOCX]

Table S9: Selected SSR data verifying the non-clonal origin of synthetic Ranunculus F_2_ hybrids by depicting the presence of paternal private alleles. m, maternal; p, paternal; N, drop out. The total matrix comprises six loci with altogether 33 alleles (coded as binary presence/absence data).

|  | **LH08_164** | **R84_171** | **LH11_218** | **LH11_242** | **R2562_367** | **R2562_405** | **R2477_265** |
| --- | --- | --- | --- | --- | --- | --- | --- |
| **f1_J24_m** | 0 | 0 | 0 | 0 | 0 | 0 | 0 |
| **f1_J22A_p** | 1 | 1 | 1 | 1 | 1 | 1 | 1 |
| f2_J24xJ22_1 | 0 | 0 | 0 | 1 | 0 | 1 | 0 |
| f2_J24xJ22_10 | 0 | 1 | 1 | 0 | 1 | 0 | 1 |
| f2_J24xJ22_11 | 0 | 0 | N | N | N | N | N |
| f2_J24xJ22_12 | N | 1 | 1 | 1 | N | N | 0 |
| f2_J24xJ22_13 | 0 | 1 | N | N | N | N | N |
| f2_J24xJ22_14 | N | 0 | 1 | 0 | 0 | 1 | 1 |
| f2_J24xJ22_15 | 0 | 0 | 0 | 1 | 0 | 1 | 1 |
| f2_J24xJ22_16 | 0 | N | 1 | 1 | 0 | 0 | 0 |
| f2_J24xJ22_17 | 0 | 1 | 0 | 1 | 0 | 0 | 1 |
| f2_J24xJ22_18 | 0 | 1 | 1 | 1 | N | N | 0 |
| f2_J24xJ22_19 | 0 | 1 | 0 | 0 | 1 | 1 | 1 |
| f2_J24xJ22_2 | 0 | 1 | 0 | 1 | N | N | 1 |
| f2_J24xJ22_20 | 0 | 1 | N | N | N | N | N |
| f2_J24xJ22_21 | N | N | 0 | 0 | 0 | 0 | 1 |
| f2_J24xJ22_22 | 0 | 0 | 0 | 1 | 1 | 0 | 0 |
| f2_J24xJ22_23 | 0 | 1 | N | N | N | N | N |
| f2_J24xJ22_24 | N | N | 1 | 0 | 0 | 0 | 1 |
| f2_J24xJ22_25 | 0 | N | 0 | 1 | 1 | 0 | 1 |
| f2_J24xJ22_3 | 0 | 1 | 1 | 0 | 0 | 1 | 1 |
| f2_J24xJ22_4 | 0 | 0 | N | N | N | N | N |
| f2_J24xJ22_5 | N | 1 | 1 | 0 | 0 | 0 | 1 |
| f2_J24xJ22_6 | 0 | 1 | N | N | N | N | N |
| f2_J24xJ22_7 | 0 | 1 | 0 | 1 | 0 | 0 | 1 |
| f2_J24xJ22_8 | 0 | 1 | 0 | 1 | 0 | 0 | 1 |
| f2_J24xJ22_9 | 0 | 0 | 1 | 0 | 0 | 0 | 1 |
