# Supplementary material for: Establishment of Apomixis in Diploid F2 Hybrids and Inheritance of Apospory From F1 to F2 Hybrids of the Ranunculus auricomus Complex
Source: Front Plant Sci. 2018 Aug 3;9:1111. doi: 10.3389/fpls.2018.01111 (PMC6085428; doi:10.3389/fpls.2018.01111)
Supplement: Supplementary file 24 [file Table_10.DOCX]

Table S10: Selected SSR data verifying the non-clonal origin of synthetic Ranunculus F_2_ hybrids by depicting the presence of paternal private alleles. m, maternal; p, paternal; N, drop out. The total matrix comprises six loci with altogether 33 alleles (coded as binary presence/absence data).

|  | **LH09_206** | **R84_162** | **R2562_367** | **R2562_405** | **R2477_299** |
| --- | --- | --- | --- | --- | --- |
| **f1_J30A_m** | 0 | 0 | 0 | 0 | 0 |
| **f1_J18B_p** | 1 | 1 | 1 | 1 | 1 |
| f2_J30AxJ18A_1 | 0 | 1 | 1 | 0 | 0 |
| f2_J30AxJ18A_2 | 0 | 1 | 1 | 0 | 1 |
| f2_J30AxJ18A_3 | 0 | 1 | 0 | 0 | 0 |
| f2_J30xJ18_1 | 0 | 0 | 1 | 1 | 0 |
| f2_J30xJ18_2 | 0 | 1 | 1 | 0 | N |
| f2_J30xJ18_3 | 1 | 1 | 0 | 0 | N |
