# Supplementary material for: Establishment of Apomixis in Diploid F2 Hybrids and Inheritance of Apospory From F1 to F2 Hybrids of the Ranunculus auricomus Complex
Source: Front Plant Sci. 2018 Aug 3;9:1111. doi: 10.3389/fpls.2018.01111 (PMC6085428; doi:10.3389/fpls.2018.01111)
Supplement: Supplementary file 25 [file Table_11.DOCX]

Table S11: Selected SSR data verifying the non-clonal origin of synthetic Ranunculus F_2_ hybrids by depicting the presence of paternal private alleles. m, maternal; p, paternal; N, drop out. The total matrix comprises six loci with altogether 33 alleles (coded as binary presence/absence data).

|  | **LH08_180** | **R84_171** | **LH11_224** | **R2562_357** | **R2562_375** | **R2477_285** |
| --- | --- | --- | --- | --- | --- | --- |
| **f1_J6_m** | 0 | 0 | 0 | 0 | 0 | 0 |
| **f1_F3A_p** | 1 | 1 | 1 | 1 | 1 | 1 |
| f2_J6xF3_1 | 0 | 1 | 0 | 1 | 0 | 0 |
| f2_J6xF3_10 | 1 | 0 | 0 | 0 | 1 | 0 |
| f2_J6xF3_11 | 0 | 1 | 0 | 1 | 0 | 0 |
| f2_J6xF3_12 | 1 | 1 | N | N | N | 0 |
| f2_J6xF3_13 | 0 | 0 | 0 | 1 | 0 | 0 |
| f2_J6xF3_14 | 0 | 1 | 0 | 1 | 0 | 1 |
| f2_J6xF3_15 | 1 | 0 | 0 | 0 | 0 | 0 |
| f2_J6xF3_18 | 0 | 1 | 0 | 0 | 0 | 0 |
| f2_J6xF3_19 | 1 | 1 | 0 | 0 | 0 | 1 |
| f2_J6xF3_20 | 1 | 0 | 0 | 0 | 1 | 1 |
| f2_J6xF3_21 | 0 | 0 | 0 | 0 | 0 | 0 |
| f2_J6xF3_22 | 0 | 1 | 0 | 1 | 0 | 1 |
| f2_J6xF3_23 | 1 | 1 | 1 | 0 | 0 | 0 |
| f2_J6xF3_24 | 1 | 0 | 0 | N | N | 0 |
| f2_J6xF3_25 | 1 | 1 | 1 | 0 | 0 | 0 |
| f2_J6xF3_27 | 0 | 0 | 0 | 1 | 0 | 0 |
| f2_J6xF3_28 | 0 | 1 | 1 | 1 | 0 | N |
| f2_J6xF3_29 | 0 | 1 | 0 | 1 | 0 | N |
| f2_J6xF3_3 | 0 | 1 | 0 | 0 | 0 | 0 |
| f2_J6xF3_30 | 1 | 0 | 0 | 1 | 0 | 0 |
| f2_J6xF3_4 | 0 | 1 | 0 | 1 | 0 | 1 |
| f2_J6xF3_5 | 0 | 1 | 1 | 1 | 0 | 1 |
| f2_J6xF3_6 | 0 | 1 | 0 | 0 | 0 | 0 |
| f2_J6xF3_7 | 0 | 1 | 0 | N | N | N |
| f2_J6xF3_8 | 1 | 1 | 0 | 0 | 1 | N |
| f2_J6xF3_9 | 0 | 1 | 0 | N | N | 1 |
