# Supplementary material for: Establishment of Apomixis in Diploid F2 Hybrids and Inheritance of Apospory From F1 to F2 Hybrids of the Ranunculus auricomus Complex
Source: Front Plant Sci. 2018 Aug 3;9:1111. doi: 10.3389/fpls.2018.01111 (PMC6085428; doi:10.3389/fpls.2018.01111)
Supplement: Supplementary file 26 [file Table_12.DOCX]

Table S12: Selected SSR data verifying the non-clonal origin of synthetic Ranunculus F_2_ hybrids by depicting the presence of paternal private alleles. m, maternal; p, paternal; N, drop out. The total matrix comprises six loci with altogether 33 alleles (coded as binary presence/absence data).

|  | **R84_171** | **R84_174** | **LH11_230** | **R2562_357** | **R2562_375** | **R2477_285** |
| --- | --- | --- | --- | --- | --- | --- |
| **f1_J6_m** | 0 | 0 | 0 | 0 | 0 | 0 |
| **f1_F7A_p** | 1 | 1 | 1 | 1 | 1 | 1 |
| f2_J6xF7_1 | 0 | 1 | 0 | 1 | 0 | 0 |
| f2_J6xF7_10 | 1 | 0 | 1 | 1 | 0 | N |
| f2_J6xF7_11 | 1 | 0 | 1 | 1 | 0 | 0 |
| f2_J6xF7_12 | 1 | 0 | 1 | 1 | 0 | 0 |
| f2_J6xF7_13 | N | N | 1 | 1 | 0 | 0 |
| f2_J6xF7_15 | 1 | 0 | 1 | 1 | 0 | 0 |
| f2_J6xF7_2 | 1 | 0 | 1 | 1 | 0 | N |
| f2_J6xF7_3 | 1 | 0 | 1 | 1 | 0 | 0 |
| f2_J6xF7_4 | N | N | N | 1 | 0 | N |
| f2_J6xF7_5 | 1 | 1 | 0 | 1 | 0 | N |
| f2_J6xF7_6 | 0 | 1 | 0 | N | N | 0 |
| f2_J6xF7_7 | 1 | 1 | 0 | N | N | 0 |
| f2_J6xF7_8 | 1 | 0 | 0 | 1 | 0 | 0 |
| f2_J6xF7_9 | 1 | 1 | 0 | 1 | 0 | N |
